# Evaluation of ILEX SelfCerv for Detection of High-Risk Human Papillomavirus Infection in Gynecology Clinic Attendees at a Tertiary Hospital in South Africa

**DOI:** 10.3390/jcm10214817

**Published:** 2021-10-20

**Authors:** Teboho Amelia Tiiti, Tebogo Loraine Mashishi, Varsetile Varster Nkwinika, Kgotlaethata Aaron Molefi, Ina Benoy, Johannes Bogers, Selokela Gloria Selabe, Ramokone Lisbeth Lebelo

**Affiliations:** 1 Department of Virological Pathology, Sefako Makgatho Health Sciences University, Pretoria 0204, South Africa; tebohotiiti@yahoo.co.za (T.A.T.); mashishitebogolorraine@gmail.com (T.L.M.); vnkwinika@gmail.com (V.V.N.); gloria.selabe@smu.ac.za (S.G.S.); 2 Laboratory of Cell Biology and Histology, Faculty of Medicine and Health Sciences, University of Antwerp, 2610 Antwerpen, Belgium; ibenoy@ambior.org (I.B.); john-paul.bogers@uantwerpen.be (J.B.); 3 South African Vaccination and Immunization Centre, Sefako Makgatho Health Sciences University, Pretoria 0204, South Africa; 4 Department of Obstetrics and Gynaecology, Sefako Makgatho Health Sciences University, Pretoria 0204, South Africa; molefi62@yahoo.com; 5 Algemeen Medisch Laboratorium (AML), Sonic Healthcare, 2020 Antwerpen, Belgium; 6 Department of Anatomical Pathology, Sefako Makgatho Health Sciences University, Pretoria 0204, South Africa; 7 National Health Laboratory Service, Department of Virological Pathology, Sefako Makgatho Health Sciences University, Pretoria 0204, South Africa

**Keywords:** South Africa, SelfCerv, HPV, cervical cancer, self-sampling, E6/E7 mRNA, preference

## Abstract

Background: The SelfCerv Self-Collection Cervical Health Screening Kit (Ilex Medical Ltd., Johannesburg, South Africa) is an applicator tampon designed for self-collection of vaginal samples for the detection of human papillomavirus (HPV) deoxyribonucleic acid (DNA) and E6/E7 messenger ribonucleic acid (mRNA). The study aimed to evaluate the performance of the SelfCerv applicator tampon for the detection of hr-HPV for cervical cancer screening, and further to investigate women’s experiences and preferences regarding self-sampling. Methods: Vaginal samples were collected from 527 gynecology clinic attendees aged ≥18 years at a tertiary hospital in Gauteng Province, South Africa. Self-samples were collected using the SelfCerv kit, followed by endocervical samples collected by a healthcare professional using Cervex-Brush^®^ Combi. Participants completed a self-administered questionnaire on self-sampling experiences and preferences. Both samples were tested for 14 high-risk (hr) HPV types and E6/E7 mRNA using the Abbott RealTime HR-HPV and Aptima HR-HPV mRNA assays, respectively. Results: The overall agreement for hr-HPV typing between 527 paired samples was good (87.1%; κ =0.74) with high sensitivity (86.2%) and specificity (88.0%). HPV-16 (96.4%; κ = 0.83) had higher agreement rate than HPV-18 (96.8%; κ = 0.72) and the other 12 hr-HPVs (86.5%; κ = 0.72). Two hundred and eighty-five (285) sample pairs tested for E6/E7 mRNA showed fair agreement (70.2%; κ= 0.34). Furthermore, self-sampling was reported as comfortable (90.5%) and painless (86.7%), with 88.4% of women preferring self-collection. Conclusions: Self-collected samples had good agreement with the healthcare professional-collected samples for the detection of hr-HPV DNA and the procedure was highly preferred by women. Self-sampling using SelfCerv can be used as an alternative to healthcare professional sampling in clinic-based routine cervical cancer screening.

## 1. Introduction

Cervical cancer is one of the most preventable and treatable diseases. However, there were an estimated 604,127 new cervical cancer cases and 341,831 deaths in 2020 worldwide [1]. Several programs have been implemented to curb cervical cancer numbers. For instance, Papanicolaou (Pap) testing has significantly reduced cervical cancer incidence and mortality rates, particularly in high-income countries (HIC) [2]. However, in low- and middle-income countries (LMICs), Pap testing has been unsuccessful because of poor organization, low coverage, and lack of quality assurance [2]. To achieve cervical cancer screening coverage, the implementation of affordable and acceptable screening and treatment services must be explored [3].

Persistent infection with one of the oncogenic types of human papillomavirus (HPV), primarily types 16 and 18, is a necessary but not sufficient cause of cervical cancer [2]. The role of HPV in cervical cancer has therefore led to the development of molecular HPV tests [4]. HPV testing has a higher sensitivity than cytology for detecting high-grade precancerous lesions, and is, therefore, the best tool for cervical cancer screening [5,6]. Moreover, unlike Pap smear, HPV testing enables women to self-collect cervicovaginal samples that may improve cervical cancer screening attendance [7].

A self-collected sample is an easy to perform, preferred, and comfortable sampling method that is increasingly adopted for the detection of HPV [8,9]. Several studies have reported the advantages of self-collection as being less costly, noninvasive, and well-accepted [10,11,12,13]. Compared to healthcare professional-collected samples, self-collected samples for HPV detection have a concordance of 89.2–94.2% [8,9], reliability (measured using the kappa statistic [κ]) ≥ 0.7 [8,9,14], sensitivity ≥ 80% and specificity > 90% [8,9,13]. Using the hr-HPV assay (polymerase chain reaction), self-samples have similar sensitivity; however, they have slightly less specificity compared to healthcare professional-collected samples [15]. There are several self-collection devices, such as brushes, swabs, and lavages, which have been developed and clinically tested for HPV testing in cervical cancer screening. The SelfCerv (Ilex Medical Ltd., Johannesburg, South Africa) applicator tampon is an improved device for discreet, easy use and handling to avoid contamination during use. Several studies have shown that the application of self-sampling devices has increased cervical cancer screening attendance and coverage [16,17,18]. Moreover, a meta-analysis in 2018 reported higher response rates for hr-HPV testing when using self-sampling kits [15]. Therefore, self-collection can be an alternative method to encourage attendance in cervical cancer screening programs. Hence, in South Africa, the National Department of Health (NDoH) Cervical Cancer Prevention and Control Policy recommends the investigation of self-sampling as a tool to increase cervical cancer screening coverage in several settings, particularly among harder to reach populations [19].

This study aimed to evaluate the performance of the SelfCerv Self-Collection Cervical Health Screening applicator tampon compared to health professional-collected samples for the detection of hr-HPV for cervical cancer screening, and further, to investigate women’s experiences and preferences regarding self-sampling versus healthcare professional sampling.

## 2. Materials and Methods

### 2.1. Study Design and Population

This was a cross-sectional study conducted at a tertiary hospital in Gauteng Province, South Africa, between 2016 and 2018. Paired samples were collected from 527 women who were enrolled in the study following obtaining written informed consent. Samples were sent to the HPV and STIs Training Centre for Africa at the Department of Virology laboratory for testing. Women aged 18 years and older were included in the study, and those who had undergone a complete hysterectomy or were going through their monthly menstrual cycle were excluded.

### 2.2. Specimen Collection

Participants received verbal instructions on how to collect the self-sample using a SelfCerv Self Collection Cervical Health Screening Kit (Ilex Medical Ltd., Johannesburg, South Africa). The SelfCerv package included pictorial diagrams and instructions for use. The SelfCerv Kit includes an applicator tampon, a patient information sticker, and a specimen bottle containing 4 mL of saline buffer. While waiting to be attended to at the clinic, consenting participants were individually taken to a private room and asked to insert the applicator tampon into the vagina until it met with resistance, and then remove the applicator and return to the waiting room. Participants then completed part 1 of the questionnaire. After tampon insertion for two hours, as specified by the manufacturer, the participants were requested by the healthcare professional to remove the tampon, taking care not to touch the tampon with their hands. The tampon has a cord/string that was gently pulled to remove it from the vagina and place it in a specimen bottle containing the saline buffer, making sure that the cord/string remained outside the bottle. To avoid contamination, there was no direct contact of the tampon with the hands during insertion and removal of the tampon. The healthcare professional then examined the participants and obtained an endocervical sample using a Cervex-Brush^®^ Combi (Rovers Medical Devices, B.V., Oss, The Netherlands) with the assistance of a speculum. The collection brush containing endocervical cells was immediately rinsed into a ThinPrep PreservCyt solution vial (Hologic Incorporated, Bedford, MA, USA) and then discarded. Participants were then requested to complete the self-sampling part of the questionnaire. Both samples were placed in a medical box and transported to the laboratory for processing. Upon arrival, 20 mL of ThinPrep PreservCyt solution was added to the specimen bottle containing the tampon. The bottle was swirled and then squeezed to remove the tampon from the bottle. The solution from the specimen bottle was transferred into an empty ThinPrep vial and the vial was labeled properly.

### 2.3. Data Collection

The questionnaire consisted of four main parts. The first part of the questionnaire collected demographic data including age, marital and employment status, place of residence, race, sexual practices, and reproductive history. The second and third parts included questions on HPV and cervical cancer. The fourth part included six closed-ended (Yes/No) questions to evaluate the participant’s experiences and preferences using the self-sampling device. Participants were asked to fill in part 4 of the questionnaire, which was on self-sampling experiences and preferences after they had self-collected and a healthcare professional had taken the sample. The questions to assess self-sampling experiences and preferences were: *“Were you comfortable using the self-collection device?”, “Did you experience any pain when self-collecting the sample?”, “Would you prefer to self-collect the sample for cervical cancer screening in the future?”, “Would you prefer to use the self-collection device to collect a sample for cervical cancer screening in the future?”, “Would you prefer the healthcare professional to take the sample for cervical cancer screening”?, “Are you worried that you have not collected the sample properly?”*. The data were double entered into Microsoft Excel.

### 2.4. Analysis of Samples for the Presence of High-Risk HPVs

HPV genotyping was performed using Abbott RealTime High-Risk (HR) HPV assay (Abbott Molecular GmbH & Co. KG, Wiesbaden, Germany) according to the manufacturer’s instructions. A volume of 0.8 mL of self-collected and healthcare professional-collected samples from each ThinPrep PreservCyt vial was transferred into Abbott Master Mix tubes and labeled properly. This assay detects 14 high-risk HPV types (16, 18, 31, 33, 35, 39, 45, 51, 52, 56, 58, 59, 66, and 68) and only genotypes HPV-16 and HPV-18 from the other 12 hr-HPV types. Probes for HPV-16 and HPV-18, “other hr-HPV” types (31, 33, 35, 39, 45, 51, 52, 56, 58, 59, 66, and 68) and IC were labeled with four fluorophores allowing their signals to be distinguishable in a single reaction to differentiate HPV-16 and HPV-18 from other 12 hr-HPVs. The HPV target cutoff (32.00 cycle threshold (Ct)) as well as the internal control target cutoff (35.00 Ct) is already established by the manufacturer. Samples with insufficient content of cervical samples are automatically invalidated. The endogenous human beta-globin sequence was detected as sample validity control for cell adequacy

### 2.5. Analysis of Samples for HPV E6/E7 mRNA

The Aptima HPV mRNA assay (Hologic Gen-Probe, Inc., San Diego, CA, United States) was used to detect E6/E7 viral mRNA from 14 hr-HPV types (16, 18, 31, 33, 35, 39, 45, 51, 52, 56, 58, 59, 66 and 68). Briefly, an aliquot of 1 mL was transferred into the Aptima Specimen Transfer Tube containing specimen transport media that lyses the cells and releases the mRNA and protects it from degradation. During testing, the mRNA was isolated from the specimen using capture oligomers that are linked to magnetic microparticles. Sequences complementary to specific regions of the HPV mRNA target molecules and a string of deoxyadenosine residues were contained in the capture oligomers. Sequence-specific regions of the capture oligomers bind to specific regions of the HPV mRNA target molecule during the hybridization step. HPV mRNA was amplified using transcription-based nucleic acid amplification and detection was achieved by Hybridization Protection Assay. Assay results were interpreted on the basis of the signal/cutoff ratio for the analyte, and specimens with signal/cutoff ratios of ≥0.5 were considered positive.

### 2.6. Data Analysis

Data analysis was performed using STATA version 14.1 (Stata Corp., College Station, TX, USA). Results of categorical data were presented as frequencies and percentages. For continuous data (age), mean and standard deviation (SD) were calculated. The overall agreement (percent agreement, kappa values with 95% confidence intervals) was determined using the kappa (κ) statistic. Kappa values were considered as poor (≤0.20), fair (0.21–0.4), moderate (0.41–0.60), good (0.61–0.80), and very good (0.81–1.00). Healthcare professional-collected test results were used as a reference standard to estimate the sensitivity and specificity of the applicator-tampon collection method.

## 3. Results

### 3.1. Demographic Characteristics of Study Participants

The mean (±standard deviation (SD)) age of participants was 36.8 (SD: ±11.0; range 18–68) years. Most (32.6%) of the participants were between the ages of 30 and 39, followed by those aged less than 30 years (28.7%). The majority (75.9%) of the participants were either single and/or divorced/widowed/separated, 54.8% were unemployed, and 85.0% were from semi-urban areas. Most (99.6%) of the participants were black Africans and 52.8% reported never being screened for cervical cancer before the study. The majority (44.2%) of the participants attended the clinic for routine Pap smear with the least (5.7%) of the participants seeking family planning services. The study population included 66.4% of women who were asymptomatic (Table 1).

### 3.2. Performance of HPV Self-Sampling Relative to Healthcare Professional Sampling

DNA extraction and amplification was successfully achieved on all paired samples, as indicated with a positive internal control. For self-collected samples, the overall HPV prevalence was 47.6% (251/527); for healthcare professional-collected samples, 48.0% (253/527). HPV-16 in self-collected samples and healthcare professional-collected samples were detected in 63 (12.0%) and 62 (11.7%), respectively. Self-collection detected 6.5% (34/527) and 42.1% (222/527) for HPV-18 and other 12 hr-HPVs compared to 5.9% (31/527) and 41.9% (221/527) by healthcare professional sampling. The results for hr-HPV DNA and HPV E6/E7 mRNA testing were obtained for 527 and 285 participants, respectively (Table 2). Overall, the percent agreement for detection of hr-HPV DNA was 87.1%, and the Cohen (κ) coefficient was 0.74 for agreement between self-and healthcare professional-collected samples. Compared with the healthcare professional-collected samples, self-collected samples demonstrated high sensitivity (86.2%) and specificity (88.0%) for the detection of overall hr-HPV DNA, respectively. Overall, there were 68 discordant samples, 33 were negative by healthcare professional sampling, but positive by self-sampling, and 35 were negative by self-sampling, but positive by healthcare professional sampling. For HPV-16, the agreement was very good (96.4%; κ = 0.83) compared to HPV-18 (good; (96.8%; κ = 0.72)). Self-samples for the detection of HPV-16 demonstrated high specificity (97.9%). The sensitivity (77.4%) was lower in self-collected samples for HPV-18 detection but had comparable specificity (98.0%) to that for HPV-16 (97.9%). Other 12 hr-HPVs detection in self-collected samples had a concordance of 86.5% (κ = 0.72). For the other 12 hr-HPVs, the sensitivity and specificity were 84.2% and 88.2%, respectively. For HPV E6/E7 mRNA, self-collected samples demonstrated a fair level of agreement (70.2%; κ = 0.34), sensitivity (68.3%), and specificity (77.1%).

### 3.3. Discordant hr-HPV Test Results

There were 19 disagreements between self and healthcare professional HPV tests for HPV-16, 17 disagreements for HPV-18, and 71 for other 12 hr-HPVs. Of all disagreements, 56/107 (52.3%) results were positive for self-collected samples and negative for the healthcare professional-collected samples. No follow-up is available for these women. Discrepant self-collected test results were positive at high cycle threshold values (Table 3).

### 3.4. Women’s Experience and Preference for Self-Sampling

A total of 526 out of 527 participants answered the questionnaire. Self-sampling using the SelfCerv was recorded as comfortable by 90.5% while 13.3% recorded experiencing pain. Most participants reported that they would prefer to use the SelfCerv self-sampling device (88.4%) or self-collect the sample (87.1%) regardless of the device. A total of 402 (76.4%) participants recorded that they would also prefer the healthcare professional to collect the sample. Very few participants (24.3%) were worried that they did not collect the sample properly (Table 4).

### 3.5. Women’s Experience and Preference for Self-Sampling by Age Group and Screening

As shown in Table 5, women aged 40–49 and 50–59 years were more likely to be comfortable with using the self-collection device than the other age groups. Comfortability using the SelfCerv device was statistically associated with age (*p* = 0.007). The results further show that women aged 40–49 years were more likely to prefer the healthcare professional to collect the sample. Women in the age group 60 years and older were less worried that they had not collected the sample properly but more likely to have experienced pain when self-collecting the sample. A low proportion (12.6%) of participants who have never been screened before the study recorded not being comfortable using the self-collection device. Most unscreened participants (86.0%) recorded not having experienced pain during self-collection. The majority (89.9%) of participants who had never been screened recorded that they would prefer to use the self-collection device (SelfCerv) for future cervical cancer screening, as opposed to 28 (10.1%) who answered “no” to the question.

## 4. Discussion

Interventions to increase cervical cancer screening coverage are important. This is supported by the data from the current study, which show that the majority of women in the current study had never been screened for cervical cancer using the Pap test at the time of the study. The reasons why these women had never been screened were not explored. In HPV self-sampling, sample validity depends on the participants; hence, it is important to compare HPV test results from self-collected samples with those collected by a healthcare professional, which are considered the gold standard.

The current study revealed 87.1% concordance between self-and healthcare professional-collected samples in detecting overall hr-HPV with κ 0.74, which showed good agreement. The level of agreement in our study was not as high as that in a study in Ghana among 194 women using the careHPV brush coupled with the careHPV assay [8]. Other studies revealed a similar level of agreement as in our study; κ: 0.75 [20], κ: 0.74 [21], and κ: 0.71 [22]. However, other studies in Ethiopia (κ: 0.58) [23] and Cameroon (κ: 0.52) [24] reported a lower level of agreement compared to our study. It is uncertain if these differences reflect the different self-sampling devices used, HPV testing methods, population, and/or sample size. Regarding false-positive results by self-sampling, the tampon might have picked up vulvovaginal HPV infection. Regarding individual HPV types, the level of agreement for HPV-16 was very good, with a concordance of 96.4% and κ: 0.83 compared to HPV-18 (96.8%; κ = 0.72) and the other 12 hr-HPV (86.5%; κ: 0.72). It might be that HPV-16 is present in higher loads compared to the other types and therefore easily recoverable even following DNA loss during transportation, processing, and storage. The level of agreement for HPV-16 and HPV-18 in our study is consistent with a South African study that used non-applicator tampons and demonstrated a very good (κ: 0.88) and good (κ: 0.80) level of agreement, respectively [25]. However, the level of agreement for HPV-16 and HPV-18 in our study was high compared to a previous study that reported κ: 0.72 and κ: 0.48 for HPV-16 and HPV-18, respectively [26].

For overall hr-HPV, the sensitivity and specificity of self-collected samples compared with healthcare professional-collected samples were 86.2% and 88.0%, respectively. Similar findings were reported in a study conducted among women with reproductive tract infections using Roche Reverse Line Blot Assay [25]. In Malawi, Esber et al. [13] in 2018 reported a lower sensitivity (79%); however, they also reported higher specificity (99%) compared to our findings. In our study, the sensitivity of self-collected samples for HPV-16 (85.5%) was higher than 75%, and the specificity (97.9%) was comparable to the 100% reported in a previous study [13]. The sensitivity and specificity for the other 12 hr-HPVs in the current study were similar to that previously reported in Japan [27].

There are limited data on the performance of self-collected samples for hr-HPV mRNA detection. In the current study, the SelfCerv applicator tampon coupled with Aptima HR-HPV mRNA assay demonstrated a fair level of agreement (70.2%; κ: 0.34). In South Africa and Kenya, Adamson et al. [28] and Senkomago et al. [29] found 77.6–82.8% concordance with κ: 0.54–0.55, which represented moderate agreement between these two methods. Adamson et al. [28] reported high sensitivity (77.4%); however, they reported similar specificity (77.4%) compared to the current study. Arbyn et al. [30], in a meta-analysis, reported that self-samples were less sensitive and specific with signal-based assays. Toliman et al. [31] also reported low sensitivity of self-collected samples using the Aptima HR-HPV assay. Furthermore, in a recent meta-analysis, Arbyn et al. [15] reported that mRNA testing using Aptima was less sensitive but as specific in self-collected samples. These results may be due to the sampling location, as the speculum-assisted health professional samples are collected from the cervix directly in the transformation zone, whereas the applicator tampon collects a mix of exfoliated cervical and vaginal cells. The self-collected samples may not have a substantial number of cells from the transformation zone that are infected and have a replicating virus.

In the current study, the majority of participants were comfortable self-collecting the sample using the SelfCerv applicator tampon. Consistent with previous studies, participants reported self-sampling as being painless when using nylon-tipped flocked swabs and flocked swabs [32,33] and comfortable when using self-sampling devices such as Evalyn brushes, flocked swabs, and Dacron swabs [9,33,34]. However, there were conflicting findings, since some of the participants who had reported self-sampling using the applicator tampon being comfortable but also reported experiencing pain. In the current study, participants between the ages 40–59 were more comfortable using the SelfCerv device for collection compared to the other groups. The painful and uncomfortable experience reported by the minority of participants in our study might be explained by the physical characteristics of the applicator tampon. The tampon has a cardboard applicator that allows women to push the tampon inside the vagina; the applicator may contribute to discomfort and pain, particularly if an individual is not familiar with the device and thus unable to insert it properly. In older women, the vaginal lining thins and dries out; this might explain the pain experienced by women 60 years and older in the study. Among participants who had never been screened for cervical cancer before, there was a positive response towards the self-sampling experience, as the majority showed interest to use the device for future screening.

Although a high proportion of participants preferred the self-sampling method and were willing to self-sample again, some participants were still worried about having correctly collected the sample, which is a common concern that has previously been reported [13,34]. Despite the high number of participants who preferred the self-sampling method, participants still indicated that they also preferred the samples to be collected by a healthcare professional. This could be because participants were not asked to choose one preferred method over the other. Although participants were confident and found self-sampling painless, they still preferred a more experienced individual (i.e., a healthcare professional) to collect the samples. Although the NDoH in South Africa recommends self-sampling, particularly to increase coverage among harder to reach populations, the SelfCerv might not be an ideal device for non-clinic-based settings considering the logistics of transporting the device from the point of collection to the testing laboratory. Domque et al. [35], in 2020, among women from remote villages in Cameroon reported a feasible and appropriate population-based test-and-treat cervical screening strategy using self-sampling for HPV testing. The study took an approach of having health promoters and women’s health program staff visit rural villages to educate women about self-sampling and HPV testing, whereby women provided a self-sample that was thereafter taken to the laboratory for testing [35]. Therefore, the feasibility, effectiveness, and cost-effectiveness of the SelfCerv should further be investigated in the context of screening, particularly among harder to reach populations.

### Limitations

Since only 285-paired samples were tested, this could have affected the results. The questionnaire employed in the study did not include a question assessing the educational level of participants, nor did it assess any difficulties experienced during the insertion and/or removal of the tampon.

## 5. Conclusions

The study shows that self-sampling using an applicator tampon was comparable to healthcare professional-collected samples in detecting hr-HPV DNA using the Abbott hr-HPV assay. However, the self-collected samples demonstrated a fair level of agreement for HPV E6 and E7 mRNA. Furthermore, we report a positive experience and a high preference for clinic-based self-sampling using the applicator tampon, which could be a way to increase primary screening coverage among women with limited access to screening services. The findings of the study must be interpreted with caution, as the SelfCerv might not be transferable to “harder-to-reach” populations who are often underscreened or not screened at all. Further research is needed to evaluate the effectiveness of applicator tampon-based self-collection and HPV testing for true cervical disease.

## Figures and Tables

**Table 1:** Demographic characteristics of the study participants (*n* = 527).

Variables	*n*	%
Age in years		
18–29	151	28.7
30–39	172	32.6
40–49	121	23.0
50–59	61	11.6
≥60	19	3.6
Unspecified	3	0.6
Marital Status		
Single	354	67.2
Married	126	23.9
Divorced/Widowed/Separated	46	8.7
Unspecified	1	0.2
Employment Status		
Employed	237	45.0
Unemployed	289	54.8
Unspecified	1	0.2
Place of residence		
Rural	12	2.3
Semi-Urban	448	85.0
Urban	63	12.0
Unspecified	4	0.8
Race		
African	525	99.6
Other	2	0.4
Pap screening before		
Screened	248	47.1
Not screened	278	52.8
Unspecified	1	0.2
Reason for visit		
Family planning	30	5.7
Routine Pap smear	233	44.2
Termination of pregnancy	87	16.5
Colposcopy	33	6.3
LLETZ	87	16.5
Review with Pap smear after LLETZ	57	10.8

Asymptomatic: Family planning; Routine Pap smear; Termination of pregnancy.

**Table 2:** Performance of hr-HPV and HPVE6/E7 mRNA self-sampling relative to healthcare professional sampling.

Self-Collection	Healthcare Professional Collection			% Agreement	Sensitivity (95% CI)	Specificity (95% CI)	κ Statistics (95% CI)
**Overall hr-HPV** **DNA**	Positive	Negative	Total	87.1	86.2 (81.3–90.2)	88.0 (83.5–91.6)	0.74 (0.68–0.80
Positive	218	33	251				
Negative	35	241	276				
Total	253	274	527				
**HPV-16**	Positive	Negative	Total	96.4	85.5 (74.2–93.1)	97.9 (96.1–99.0)	0.83 (0.75–0.90)
Positive	53	10	63				
Negative	9	455	464				
Total	62	465	527				
**HPV-18**	Positive	Negative	Total	96.8	77.4 (58.9–90.4)	98.0 (96.3–99.0)	0.72 (0.59–0.85)
Positive	24	10	34				
Negative	7	486	493				
Total	31	496	527				
**Other 12 hr-HPVs**	Positive	Negative	Total	86.5	84.2 (78.7–88.7)	88.2 (84.1–91.6	0.72 (0.66–0.78
Positive	186	36	222				
Negative	35	270	305				
Total	221	306	527				
**HPV E6/E7 mRNA**	Positive	Negative	Total	70.2	68.3 (61.8–74.3)	77.1 (64.5–86.9)	0.34 (0.22–0.46)
Positive	153	14	167				
Negative	71	47	118				
Total	224	61	285				

HR-HPV: high-risk human papillomavirus.

**Table 3:** Comparison of Abbott m2000 hr-HPV test results using paired self- and healthcare professional-collected samples.

HPV Type	No. of Disagreements between Self-Collected and Healthcare Professional-Collected Test Results	No. of Disagreements for Which Self-Collected Vaginal Test Result Was Positive and Healthcare Professional-Collected Sample Test Result Was Negative (%)	Mean Cycle Threshold (ct) Where Self-Collected Sample Test Result Was Positive and the Healthcare Professional Test Result Was Negative
HPV-16	19	10 (52.6)	24.43
HPV-18	17	10 (58.8)	26.45
Other 12 hr-HPVs	71	36 (50.7)	25.85

**Table 4:** Experience and preferences of self-sampling (*n* = 526).

Category	*n*	%
Were you comfortable using the self-collection device?		
Yes	476	90.5
No	50	9.5
Did you experience any pain when self-collecting the sample?		
Yes	70	13.3
No	456	86.7
Would you prefer to self-collect the sample for cervical cancer screening in the future?		
Yes	458	87.1
No	68	12.9
Would you prefer to use the self-collection device to collect a sample for cervical cancer screening in the future?		
Yes	465	88.4
No	61	11.6
Would you prefer the healthcare professional to take the sample for cervical cancer screening?		
Yes	402	76.4
No	124	23.6
Are you worried that you have not collected the sample properly?		
Yes	128	24.3
No	398	75.7

**Table 5:** Experience and preferences of self-sampling by age group and screening.

Category		Age (Years)					
		<30	30–39	40–49	50–59	60+	*p*-Value
Were you comfortable using the self-collection device?	Yes	132 (87.4)	152 (88.9)	113 (93.4)	61 (100)	16 (84.2)	0.007
	No	19 (12.6)	19 (11.1)	8 (76.6)	-	3 (15.8)	
Would you prefer to self-collect the sample for cervical cancer screening in the future?	Yes	132 (87.4)	149 (87.1)	103 (85.1)	55 (90.2)	17 (89.5)	0.932
	No	19 (12.6)	22 (12.9)	18 (14.9)	6 (9.8)	2 (10.5)	
Would you prefer the healthcare professional to take the sample for cervical cancer screening?	Yes	32 (21.2)	39 (22.8)	35 (28.9)	14 (22.9)	4 (21.0)	0.658
	No	119 (78.8)	132 (77.2)	86 (71.1)	47 (77.1)	15 (79.0)	
Are you worried that you have not collected the sample properly?	Yes	43 (28.5)	43 (25.1)	24 (19.8)	15 (24.6)	2 (10.5)	0.329
	No	108 (71.5)	128 (74.9)	97 (80.2)	46 (75.4)	17 (89.5)	
Did you experience any pain when self-collecting the sample?	Yes	26 (17.2)	23 (13.4)	13 (10.7)	4 (6.6)	4 (21.0)	0.174
	No	125 (82.8)	148 (86.6)	108 (89.3)	57 (93.4)	15 (79.0)	
Would you prefer to use the self-collection device to collect a sample for cervical cancer screening in the future?	Yes	134 (88.7)	149 (87.1)	108 (89.3)	54 (88.5)	18 (94.7)	0.946
	No	17 (11.3)	22 (12.9)	13 (10.7)	7 (11.5)	1 (5.3)	
**Category**		**Pap screening**					
		Screened	Never screened				*p*-value
Were you comfortable using the self-collection device?	Yes	233 (94.00)	243 (87.4)				0.011
	No	15 (6.0)	35 (12.6)				
Would you prefer to self-collect the sample for cervical cancer screening in the future?	Yes	214 (86.3)	244 (87.8)				0.614
	No	34 (13.7)	34 (12.2)				
Would you prefer the healthcare professional to take the sample for cervical cancer screening?	Yes	193 (77.8)	209 (75.2)				0.476
	No	55 (22.2)	69 (24.8)				
Are you worried that you have not collected the sample properly?	Yes	49 (19.8)	79 (28.4)				0.021
	No	199 (80.2)	199 (71.6)				
Did you experience any pain when self-collecting the sample?	Yes	31 (12.5)	39 (14.0)				0.606
	No	217 (87.5)	239 (86.0)				
Would you prefer to use the self-collection device to collect a sample for cervical cancer screening in the future?	Yes	215 (86.7)	250 (89.9)				0.247
	No	33 (13.3)	28 (10.1)				

## Data Availability

The data analyzed in this study is available upon request from the corresponding author.
